# Asthma hospitalization in Brazil: how much was it cost in the last fifteen years?

**DOI:** 10.1186/1939-4551-8-S1-A38

**Published:** 2015-04-08

**Authors:** Carolina Souza-Machado, Ana Carla Carvalho Coelho, Marrone Silva Lima

**Affiliations:** 1 Universidade Federal DA Bahia (Federal University of Bahia), Brazil

## Background

Asthma presents high burden in health system worldwide, including Brazil. The aim of this study was to evaluate the cost spent due to asthma hospitalization in Brazil (1998-2012).

## Methods

Data were obtained from the National Mortality Database from The Ministry of Health of Brazil. Total cost spent per year due to asthma hospitalization, media of cost per hospitalization, media of days for each event for each region were evaluated for the period 1998 to 2012 using simple descriptive analysis and times series analysis.

## Results

We recorded a total cost up to 660 million dollars in the whole period of study, from asthma hospitalization. The annual cost in Brazil is about 44 million dollars/year, which represents about 164.13 dollars for each event. Duration of a hospitalization in our country means 3,2 days per event, varying from 2,7 to 4,4 days. These costs varied from USD 50,476,829 in 2000 to USD 34,369,568 in 2012. Comparing the Brazilian regions, only at extreme years 1998 and 2012, the total cost due to asthma hospitalization were, respectively: Midwest (USD 3,246,138 and USD 3,837,184), South (USD 7,726,774 and USD 5,097,261) Southeast (USD 11,330,123 and USD 8,697,415), North (USD 2,762,194 and USD 3,837,184) and Northeast (USD 14,638,311 and USD 14,246,062). Difference between first and last years of analysis suggests decline at cost in all Brazilian regions, except North (one of the poorest region in Brazil). Furthermore, Northeast region presents the biggest spent with asthma hospitalization in Brazil (25,3% of total costs).

## Conclusions

Cost due to asthma hospitalization in Brazil is still large and unacceptable. However, a trend of slight decline was observed except in North region. Clinical implications: Focused health policies for asthma can reduce morbidity and mortality due to this disease and, consequently, reduces direct costs and burden of asthma.

